# Validity and reliability of the Greek version of the American Shoulder and Elbow Surgeons Standardized Shoulder Assessment Form

**DOI:** 10.1016/j.jseint.2020.11.007

**Published:** 2021-01-17

**Authors:** Konstantinos E. Tolis, Antonis A. Galanos, Emmanouil M. Fandridis, Konstantinos C. Soultanis, Ioannis K. Triantafyllopoulos

**Affiliations:** a Laboratory for the Research of Musculoskeletal System (LRMS), School of Medicine, National & Kapodistrian University of Athens, KAT General Hospital, Athens, Greece; b Hand, Upper limb and Microsurgery Department, KAT General Hospital, Athens, Greece; c 1st Department of Orthopaedics, School of Medicine, National & Kapodistrian University of Athens, ATTIKO General Hospital, Athens, Greece; d 5th Orthopaedic Department, HYGEIA Private Hospital, Athens, Greece

**Keywords:** ASES questionnaire, DASH score, Validity, Reliability, Greek version

## Abstract

**Background:**

The aim of our study is to prove the validity and reliability of the Greek translated version of the self-report section of the American Shoulder and Elbow Surgeons (ASES) questionnaire.

**Methods:**

A total of 108 patients with various shoulder disorders were evaluated at two different orthopedic centers. All patients answered the Greek ASES questionnaire as well as the previously validated Greek version of the Disability Arm Shoulder and Hand score. Three days after the first evaluation, a subgroup of 40 individuals was randomly selected to complete again the Greek ASES text to prove its reliability, after the test-retest procedure. Reliability was tested with Cronbach’s alpha, stability by calculating the intraclass correlation coefficient and by Blant Altman plot and structural validity with the confirmatory factor analysis.

**Results:**

The internal consistency of the ASES functional score and the ASES total score was 0.925 and 0.750 respectively. The intraclass correlation coefficient between initial assessment and reassessment of the ASES functional and total score was 0.951 and 0.938 (*P* < .001), respectively. The correlation coefficients correlation between the ASES functional and total scores with DASH total score were −0.881 and −0.759 (*P* < .001), respectively.

**Conclusions:**

The Greek ASES version proved to be equivalent to the English original version in evaluating different shoulder disorders in the Greek population.

The shoulder joint reveals a great complexity in function owing to its special anatomy and its wide and multiplane range of movement. In addition, its vicinity to the cervical spine and the brachial plexus makes the differential diagnosis of shoulder pathology more difficult. Documenting the onset of a shoulder disorder and evaluating its progress by avoiding the use of repeated laboratory or imaging techniques, seem to be crucial for both the patient and the physician.^14^ More than 30 measurements assessing shoulder function and symptoms can be detected in English literature.^1^ Many of them evaluate the general health condition, the general disabilities, and social conditions, having no sensitivity to detect changes during different stages of a certain shoulder disease. Currently, the trend is toward sensitive tools with the potential to connect dysfunction and symptoms to a certain diagnosis. A variety of self-assessment questionnaires have been published and used in orthopedic research, investigating or comparing the outcomes of conservative or surgical treatments regarding multiple shoulder pathologies. The American Shoulder and Elbow Surgeons (ASES) questionnaire has gained universal acceptance for its reliability, validity, and responsiveness as an outcome tool.^16^^,^^19^^,^^20^ Since its publication in 1994 from the Research Committee of the ASES, it has become a unique tool in evaluating the functionality of the shoulder. As a result, it has been translated and adopted in many languages.^18^^,^^24^

Many Greek publications on shoulder research refer to ASES because it is considered a joint-specific instrument.^11^ The purpose of this study was to perform the cross-cultural adaptation of the self-report section of the ASES questionnaire and to demonstrate the reliability and validity of the ASES among Greek-speaking patients with shoulder disorders.

## Methods

### Translation and cultural adaptation

Three independent bilingual (Greek as a native language and English) translators, two philologists, and one orthopedic surgeon translated the questionnaire from English to Greek. For this purpose, the cultural adaptation and translation followed the guidelines proposed by Beaton et al.^2^ The three translated manuscripts were then combined to 1 new single version, under the supervision of the authors. Reverse translation^7^ into English was then performed by a fourth independent professional translator with the English as a native language. This translator examined the text for any inconsistencies with the original English document. This validity process ensured the connectivity between the translation and the original version. The newly formed Greek prefinal version was then evaluated by a group of orthopedic experts in shoulder surgery. This prefinal questionnaire was applied to a pilot group of 15 patients, randomly chosen, suffering from a variety of shoulder pathologies. Τhey were asked to report any comments while completing the questionnaire. Their notes did not change the text, so the equivalence of the Greek version was secured. None of the aforementioned patients’ pilot group was included in the final validation study.

### Patients and data collection

The study was performed at the outpatient clinic of two different orthopedic centers (a state and a private hospital) in Athens, Greece. Inclusion criteria were (1) age ≥18 year, (2) any shoulder pathology, (3) awaiting conservative or surgical treatment, (4) no history of previous surgical intervention of the shoulder, and (5) complete ability of the patient to speak and write in Greek. From a starting pool of 140 patients, 32 were not included in the final baseline, as they left many questions unanswered. The validation analyses protocol included only 1 shoulder per patient. Every patient was assessed, while the diagnosis was confirmed by thorough orthopedic clinical examination and imaging workup.

Patients initially completed the forms in separate outpatient booths under the supervision and assistance of physiotherapists, and then, they were examined by the orthopedic surgeons.^25^ The Greek version of the ASES Subjective Form and the validated Greek version of the Disability Arm Shoulder and Hand (DASH) questionnaire were handed to the patients.^23^ The questionnaires were scored as instructed in the original ASES.

### Measurements

The ASES questionnaire consisted of 11 parts, which are divided into two sections.^20^ The first, *pain-related section,* evaluates the patient’s pain level on a 10-cm visual analog scale, setting a range from minimum “0 = no pain at all” to maximum “10 = pain as bad as it can be.” The second, *functional-related section*, contains 10 questions that evaluate the ability to perform daily activities, from simple, such as putting a coat, to more demanding such as throwing a ball overhead or lifting a 10-lbs object. Answers follow the 4-point Likert scale from “0 = unable to do” to “3 = not difficult.” Its section is assigned from 0 to 50 points, a sum of which leads from a minimum of 0 points to a maximum of 100 points. The mathematical type leading to the final ASES score is (10-visual analog scale × 5) + ((5/3) × sum of 10 questions).

The DASH questionnaire is considered a specific index, self-assessed, and independent to any pathology.^10^ It consists of 30 questions investigating the functionality of the upper extremity or symptoms deriving from orthopedic or neurologic pathologies. Four sectors are being questioned, which are symptoms, physical activity, social activity, and psychological function. Every answer is scored from a 1-5 scale, counting 1 as “no difficulty or no symptoms” to 5 as “unable to perform activity or severe symptoms.” The sum was mathematically transformed to a scale ranging from a zero (no disability) to 100 (severe disability). In this study, the previously validated Greek version of the DASH questionnaire was used.^23^

### Testing and retesting

To test the reproducibility of Greek ASES version, the questionnaires were provided twice to 40 patients at random selection. A 3-day interval was selected, as the short period of time that a shoulder pathology clinical manifestation could remain stable if left untreated. During that period, no treatment was provided to the patients, so as to avoid any clinical change. The authors evaluated the psychometric assessment by testing content validity, construct, criterion, and convergent validity of the ASES.

### Statistical analysis

*Exploratory factor analysis* using maximum likelihood extraction method with Varimax rotation was conducted for all participants to determine the factor structure of the 10 items of the ASES functional subscale. Items with factor loadings ≥40 (including values that rounded to 0.40) and those that did not load on more than 1 factor were retained. Items not meeting these criteria were removed one at a time. Factor analyses were repeated until a solution was attained in which all items included in the analysis met all criteria.^6^^,^^8^ The number factors to retain was also confirmed by using a Monte Carlo PCA.

*Confirmatory factor analysis* was used to examine and confirmed the factor structure of the questionnaire as suggested by the creator of the questionnaire. The *confirmatory factor analysis* was carried out using the Analysis of Moment Structure, version 21.0. The ASES functional consisted of 10 items, thus our sample size of 108 is within the aforementioned guidelines.

Rejecting or accepting a model was based on some global fit indices: (1) chi square–degrees of freedom ratio, (2) the root mean square error of approximation, (3) the comparative fit index, (4) the normed fit index, (5) the goodness fit index, and (6) the adjusted goodness fit index. The chi square–degrees of freedom ratio < 2.0,^21^ root mean square error of approximation < 0.08,^4^ comparative fit index >0.90,^4^ goodness fit index > 0.85,^13^ adjusted goodness fit index > 0.80,^13^ normed fit index > 0.90^3^ indicate an acceptable fit.

*Convergent or criterion validity* of the ASES questionnaire was determined by establishing its correlation to the DASH score using the Pearson’s and Spearman’s correlation coefficient. Moderate or high correlation between ASES questionnaire to the well-established DASH would support the validity of the ASES questionnaire in measuring important aspects of functional status.

*Known group’s validity* of the ASES questionnaire was examined in terms of the ability of the questionnaire to distinguish between subgroups of patients formed on the basis of their disease severity. Independent-samples t-test was used for the statistical analysis.

*Item analysis* of the ASES functional subscale was performed by analyzing the item discriminating power (corrected item correlation) and the item difficulty (item mean) depicted by the explanatory data analysis.

*Floor or ceiling effects* are considered to be present if more than 15% of respondents achieved the lowest or highest possible score, respectively.^15^

*Interpretability* refers to the degree to which one can assign qualitative meaning to quantitative scores.^4^ The error associated with a single application of the ASES was analyzed using the standard error of measurement (SEM). We estimated also the minimal detectable change (MDC). The SEM was calculated based on the formula: SEM = standard deviation x [square root (1- Cronbach alpha)]. The SEM carries with it 68% confidence interval (CI). To achieve 90% CI, the SEM was multiplied by the z value associated with the 90% CI (z = 1.65).^13^ MDC is defined as the minimal change of the ASES score that is necessary to be confident that “true change” has occurred. MDC was calculated as: MDC = standard deviation x [square root (1-intraclass correlation coefficient {ICC} value] x square root of 2. As with the error estimate, the 90% CIwas calculated by multiplying by 1.65.^13^

*A receiver operating curve analysis* was conducted to obtain the cutoff level of ASES functional and total scores for differentiation between subgroups of patients formed on the basis of their disease severity, calculating the respective areas under the curve (AUC). The areas under the *receiver operating curve* curve (AUC) with standard error and 95% CI were calculated using the maximum likelihood estimation method, and the sensitivity and specificity of different cutoff points of ASES scores were estimated using the disease severity of participants (simple-moderate vs. severe) as estimated variables.

*Internal consistency validity of the ASES* was determined by calculating Cronbach alpha coefficient.^24^ A Cronbach α coefficient value of 0.7 indicates sufficient reliability for research purposes and suggests that items are interdependent and homogeneous in terms of the construct they measure. For clinical applications a > 0.8 is desirable.^9^^,^^17^

*Test-retest reliability* indicates the stability of patients’ response in time and it was determined by calculating ICC between the initial assessment of the ASES and the reassessment after 3 days. Because this coefficient does not correct for systematic differences and agreement by chance, the scores of the 2 assessments were tested for systematic differences by using the paired t-test.^7^^,^^10^^,^^20^^,^^23^^,^^25^ The Bland-Altman plot was used as a visual method of assessing stability.

All tests were two-sided, and a *P* value of <.05 was used to denote statistical significance. All analyses were carried out using the statistical package SPSS vr 21.00 (IBM Corp., Armonk, NY, USA).

## Results

The Bartlett test of sphericity was 667.3, and it was significant (*P* < .001). The Kaiser-Meyer-Olkin Measure of Sampling Adequacy was equal to 0.931 showing suitable data for factor analysis.

The 10 items were analyzed via maximum likelihood extraction method using a varimax rotation. One factor, with eigenvalue of more than 1 and items factor loadings greater than or equal to 0≥40, was identified. The Scree test and Monte Carlo PCA for parallel analysis (the criterion value was 1.5, higher than eigenvalue of the second factor) confirmed the 1-factor solution. The eigenvalue for the first factor was 6.03, explaining 60.27% of the variance, and the eigenvalue for the second factor was 0.92, explaining only 9.2% of the variance factor loadings, which are the correlation coefficients between the items and the factor, ranged from 0.54 to 0.85 (Table I).

**Table I:** Eigenvalues and explained variance of ASES functional.

Items	Eigenvalues	% of variance	Cumulative %
**1**	**6.027**	**60.274**	**60.274**
2	0.920	9.204	69.478
3	0.624	6.245	75.723
4	0.526	5.264	80.987
5	0.433	4.331	85.318
6	0.372	3.722	89.039
7	0.370	3.697	92.736
8	0.274	2.741	95.477
9	0.237	2.369	97.846
10	0.215	2.154	100.000

*ASES*, American Shoulder and Elbow Surgeons.

Bold indicate the factor with Eigenvalue greater than 1 (just one in our case).

A single-factor model of ASES functional was conducted by confirmatory factor analysis giving acceptable global fit indices. The resulting global fit indices X^2^ = 61.88, *P* = .003, chi square–degrees of freedom ratio= 1.76, root mean square error of approximation = 0.078, comparative fit index = 0.959, normed fit index = 0.911, goodness fit index =0.887, and adjusted goodness fit index = 0.823 showed that the 1-factor solution proposed by the authors should be retained.

The correlation coefficients correlation between the ASES functional and total with DASH total score were r = −0.881 and r = −0.759 (*P* < .001), respectively. The aforementioned result indicated high correlation between two questionnaires, which satisfied the criterion validity.

The ASES functional and total scores were well discriminated between subgroups of patients on the basis of their different disease severity. The ASES functional (*P* = .001) and total scores (*P* = .019) were statistically significantly higher in participants with simple-moderate disease severity compared with those with severe DS (*P* < .05) (Table II).

**Table II:** Known-groups validity.

Variables	Disease severity	N	Mean ± SD	*P* value
ASES functional	Simple-moderate	34	30.20 ± 11.23	.001
	Severe	74	21.42 ± 12.85	
ASES total	Simple-moderate	34	55.34 ± 21.93	.019
	Severe	74	43.51 ± 24.83	

*ASES*, American Shoulder and Elbow Surgeons.

**Figure 1:**
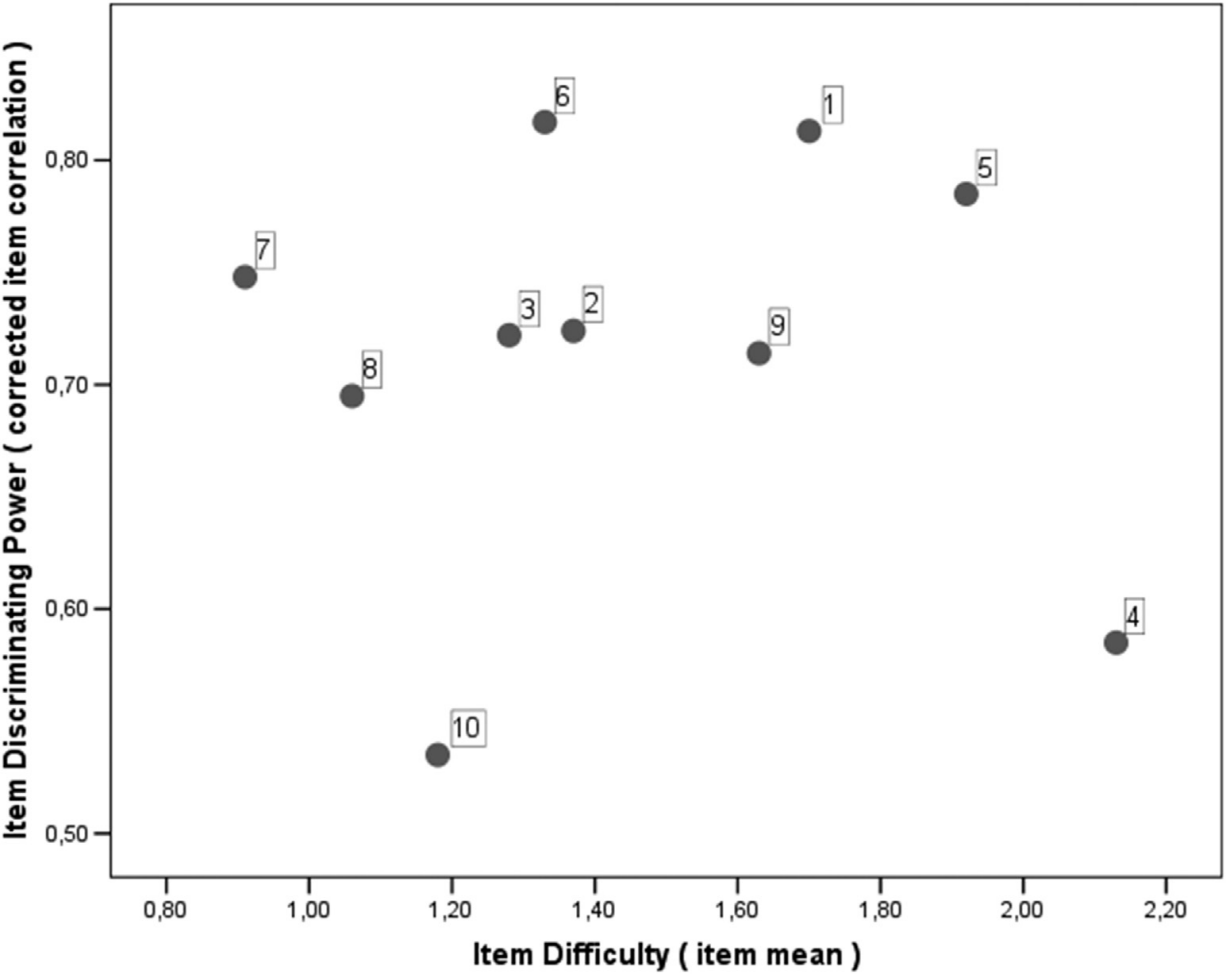
Item analysis for the function items of the ASES. ASES, American Shoulder and Elbow Surgeons.

The item analysis of the ASES showed that item 6 (*reaching a high shelf*) had the highest corrected item correlation, whereas item 10 (doing usual sport) had the lowest corrected item correlation. In addition, item 7 (lift ability) had the lowest item means and item 4(washing back) had the highest item means (Figure 1).

The percentage of patients scoring at the lowest possible level of the scale and at the highest possible level for the ASES functional and total were 1.9%, 0.9% and 0.9%, 0.9% respectively. The critical value of 15% was not surpassed so there were neither ceiling nor floor effect for the ASES questionnaire.

The error associated with the ASES functional and total at a given point in time (SEM) was ±5.8 and ±21.2 points, respectively (based on a 90% CI). The MDC for both scales at the 90% CI was ±6.7 and ±14.2 scale points, respectively.

**Figure 2:**
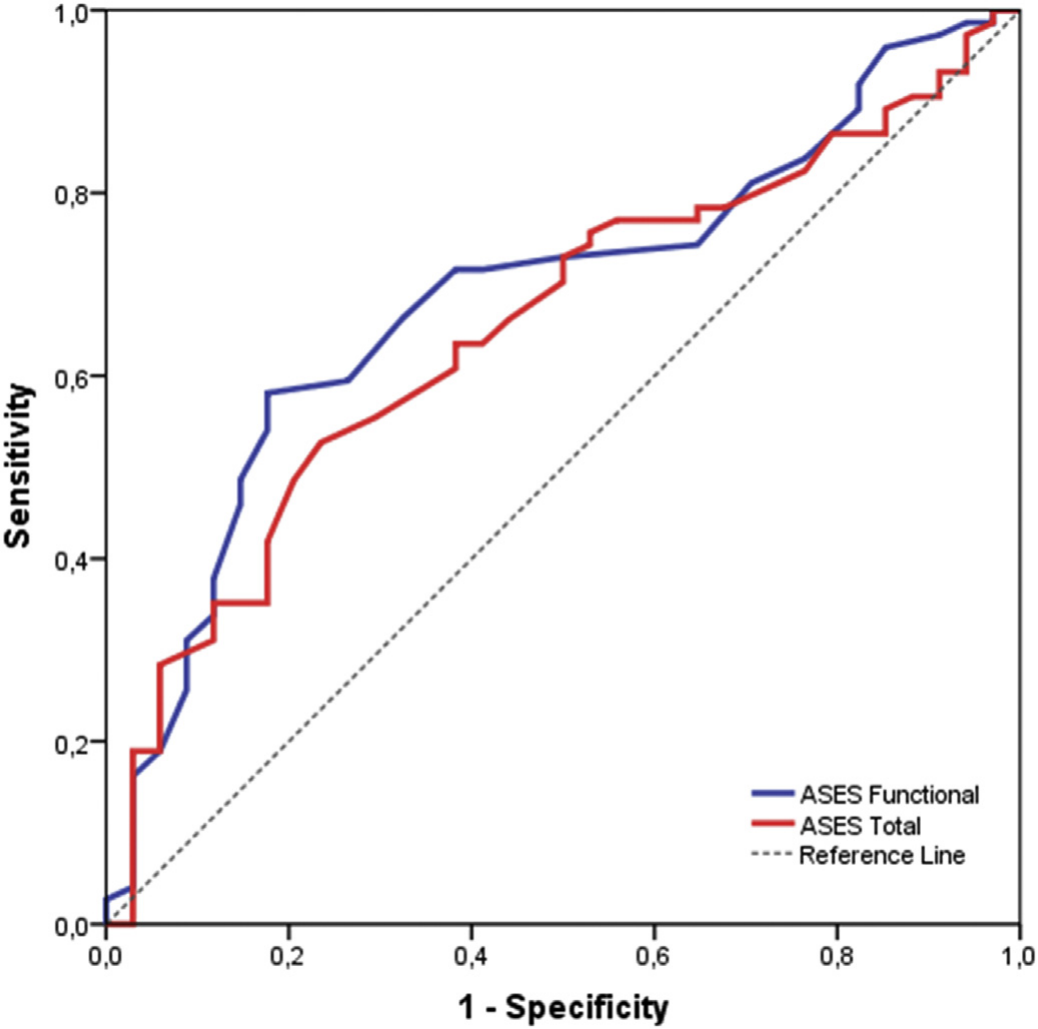
ROC analysis. ROC, receiver operating curve.

The AUC of ASES functional was 0.689 (95% CI 0.58-0.79; *P* = .002) with cutoff point 22.5, sensitivity 58%, and specificity 82%. The AUC of ASES total was 0.654 (95% CI 0.55-0.76; *P* = .05) with cutoff point 42.5, sensitivity 53%, and specificity 76% (Table III, Figure 2).

**Table III:** The cutoff point of ASES functional and total score.

Variables	AUC	*P* value	Cutoff point	Sensitivity	Specificity	95% CI
ASES functional∗	0.689	.002	22.5	58%	82%	0.585-0.793
ASES total∗	0.654	.054	42.5	53%	76%	0.547-0.761

*ASES*, American Shoulder and Elbow Surgeons; *AUC*, area under curve; *CI*, confidence interval.

∗ Smaller test result indicates more positive test.

**Figure 3:**
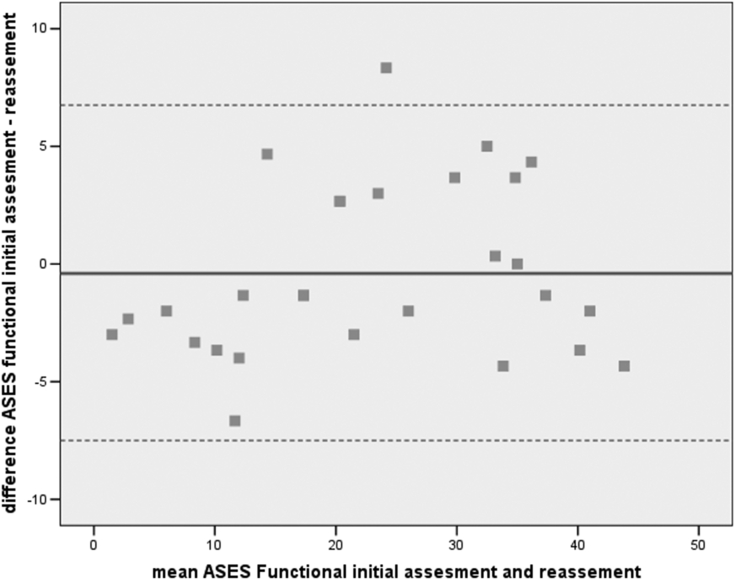
Bland-Altman plot of ASES functional. Μean difference: 0.30 (95% CI −2.11 to 2.71). *ASES*, American Shoulder and Elbow Surgeons; *CI*, confidence interval.

**Figure 4:**
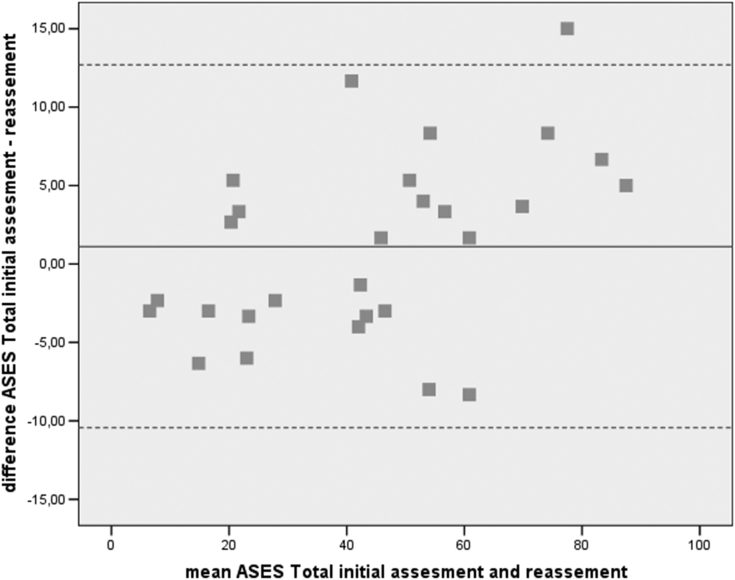
Bland-Altman plot of ASES total. Mean difference: 1.13 (95% CI −10.43 to 12.69). *ASES*, American Shoulder and Elbow Surgeons; *CI*, confidence interval.

The internal consistency of the ASES functional and total was measured with Cronbach's alpha and estimated as 0.925 and 0.750, respectively, which indicate excellent internal consistency of ASES functional score and good for ASES total score.

The paired-samples t-test between initial assessment and reassessment of ASES functional and total scores indicated no statistically significant difference. The ICC between initial assessment and reassessment of the ASES functional and total score was 0.951 and 0.938 (*P* < .001), respectively. Regarding the Bland-Altman plots, inspection of scattergram showed that almost all differences were within mean difference ±2 SDs, thus confirming the agreement between 2 assessments (Figures 3 and 4). The aforementioned results of stability indicated that ASES functional and total scores were remarkably consistent between initial assessment and reassessment (Table IV).

**Table IV:** Test-retest reliability for the ASES functional.

(N = 40)	ICC 95% CI	Paired samples t-test		*P* value
		Initial	Reassessment	
		Mean ± SD		
ASES functional	0.951∗ (0.93-0.97)	22.92 ±12.91	23.32 ± 12.11	.562
ASES Total	0.938∗ (0.92-0.96)	44.35 ± 24.53	43.21 ± 21.74	.320

*ASES*, American Shoulder and Elbow Surgeons; *CI*, confidence interval; *ICC*, intraclass correlation coefficient.

∗ *P* < .001.

## Discussion

The present study investigated the cultural adaptation and equivalence, as well as the psychometric properties of the self-administrated ASES questionnaire to the Greek language. Our results indicate that the Greek ASES version has good reliability and validity.

It has been suggested that a questionnaire should not reach a ceiling or floor value more than 15%.^9^ The Greek study had quite lower floor or ceiling effects than 15%. It is possible that the floor or ceiling effect is not present when using the Greek ASES version. Research^12^ showed that when ASES was used in variable shoulder disabilities, the results showed low percentages regarding the ceiling or floor effect in certain conditions. This implies that the ASES can be applied to many shoulder pathologies, so as to differentiate between levels of disability and clinical changes.^19^

Considering the reproductivity of the Greek version, the results were excellent. The ICC of the functional ASES was 0.951, while the total ASES reached 0.938. The results are slightly better than those previously published,^5^^,^^18^ regarding other translations of the original text for example the reproducibility ICC of the Finnish total ASES index in all patients was 0.79 (95% Cl: 0.69 to 0.86)^19^ and higher than the original ASES (0.86).^16^ Our results were further emphasized by the Bland-Altman plot, which confirmed that the differences between ASES functional and ASES total scores did not show a percentage error.

Bearing in mind that in the original version, the Cronbach alpha factor was 0.86,^16^ our study showed good internal consistency. The ASES functional was estimated at 0.925, which is considered an excellent score, while ASES total score was 0.750 being characterized as acceptable. The Greek ASES total score version ICC is lower than the Italian,^18^ the German,^22^ or the Arabic version.^26^ This result is a benefit to our study, as it implies that the items are not too homogenous, they relate reasonably, providing distinctive details about every patient.

One of the main goals of this research was to prove the correlation of the Greek ASES version to the Greek DASH version, which is widely approved and has already been validated. Previous studies showed high correlation between the ASES and other upper extremity questionnaires.^19^ The DASH score seems to be more related to Greek ASES function score than ASES total score. As the Finnish study^19^ had similar phenomena, it has been suggested that this is because of the fact that 50% of the ASES consists of single value of pain visual analog scale score and the other 50% consists of function score that has an adequate similarity to the DASH. Owing to high correlation of the referred shoulder questionnaires, the criterion validity was satisfied.

As far as the length of the Greek version of the ASES is concerned, it was considered acceptable, with the ability to be self-administrated and sufficiently understandable. It is mentioned that in the early stages of the study, it was decided during translation of the question about weight lifting 10 lbs to be adapted to the metric system as 4.5 kg, despite the fact that most studies preferred the variance of 4 to 5 kg.^18^

A limitation to our study could be the fact that all involved patients were suffering from a chronic shoulder pathology. Thus, the group examined did not represent the wider range of shoulder pathology including acute cases. However, the fact that the validation was performed by shoulder surgery specialists in two different orthopedic reference centers gives strength to the final evaluation.

## Conclusions

Based on thorough statistical analysis, the Greek ASES questionnaire proved to be as valid and reliable as the original English version. Therefore, it can be successfully applied to assess shoulder pathology among the Greek patients, irrespective of age, socioeconomic status, or diagnosis.

## Disclaimers:

*Funding:* No funding was disclosed by the author(s).

*Conflicts of interest:* The authors, their immediate families, and any research foundations with which they are affiliated have not received any financial payments or other benefits from any commercial entity related to the subject of this article.

## References

[1] Angst F., Schwyzer H.K., Aeschlimann A., Simmen B.R., Goldhahn J. (2011). Measures of adult shoulder function: Disabilities of the Arm, Shoulder, and Hand Questionnaire (DASH) and its short version (QuickDASH), Shoulder Pain and Disability Index (SPADI), American Shoulder and Elbow Surgeons (ASES) Society standardized shoulder assessment form, Constant (Murley) Score (CS), Simple Shoulder Test (SST), Oxford Shoulder Score (OSS), Shoulder Disability Questionnaire (SDQ), and Western Ontario Shoulder Instability Index (WOSI). Arthritis Care Res (Hoboken).

[2] Beaton D.E., Bombardier C., Guillemin F., Ferraz M.B. (2000). Guidelines for the process of cross-cultural adaptation of self-report measures. Spine (Phila Pa 1976).

[3] Cronbach L.J. (1951). Coefficient alpha and the internal structure of test. Psychometrica.

[4] Finch E., Brooks D., Stratford P., Mayo N. (2002). Physical rehabilitation Outcome Measures: A guide to Enhanced Clinical Decision Making.

[5] Goldhahn J., Angst F., Drerup S., Pap G., Simmen B.R., Mannion A.F. (2008). Lessons learned during the cross-cultural adaptation of the American Shoulder and Elbow Surgeons shoulder form into German. J Shoulder Elbow Surg.

[6] Gorgush R.L. (1978). Psychometric Theory.

[7] Guillemin F., Bombardier C., Beaton D. (1993). Cross-cultural adaptation of health-related quality of life measures: literature review and proposed guidelines. J Clin Epidemiol.

[8] Harman H.H. (1967). Modern Factor Analysis.

[9] Higgins P.A., Straub A.J. (2006). Understanding the error of our ways: Μapping the concepts of validity and reliability. Nurs Outlook.

[10] Hudak P.L., Amadio P.C., Bombardier C. (1996). Development of an upper extremity outcome measure: the DASH (disabilities of the arm, shoulder and hand). The Upper Extremity Collaborative Group (UECG). Am J Ind Med.

[11] Kirkley A., Griffin S., Dainty K. (2003). Scoring systems for the functional assessment of the shoulder. Arthroscopy.

[12] Kocher M.S., Horan M.P., Briggs K.K., Richardson T.R., O’Holleran J., Hawkins R.J. (2005). Reliability, validity, and responsiveness of the American Shoulder and Elbow Surgeons subjective shoulder scale in patients with shoulder instability, rotator cuff disease, and glenohumeral arthritis. J Bone Joint Surg Am.

[13] Leggin B.G., Shaffer M.A., Neuman R.M., Williams G.R., Ianotti J.P. (2003). Relationship of the Penn Shoulder Score with measures of range of motion and strength in patients with shoulder disorders: a preliminary report. Univ Pennsyl Orthop J.

[14] Luime J.J., Koes B.W., Hendriksen I.J., Burdorf A., Verhagen A.P., Miedema H.S. (2004). Prevalence and incidence of shoulder pain in the general population; a systematic review. Scand J Rheumatol.

[15] McHorney C.A., Tarlov A.R. (1995). Individual-patient monitoring in clinical practice: are available health status surveys adequate?. Qual Life Res.

[16] Michener L.A., McClure P.W., Sennett B.J. (2002). American Shoulder and Elbow Surgeons Standardized Shoulder Assessment Form, patient self-report section: reliability, validity, and responsiveness. J Shoulder Elbow Surg.

[17] Nunnally J.C. (1978). Psychometric theory.

[18] Padua R., Padua L., Ceccarelli E., Bondi R., Alviti F., Castagna A. (2010). Italian version of ASES questionnaire for shoulder assessment: cross-cultural adaptation and validation. Musculoskelet Surg.

[19] Piitulainen K., Paloneva J., Ylinen J., Kautiainen H., Hakkinen A. (2014). Reliability and validity of the Finnish version of the American Shoulder and Elbow Surgeons Standardized Shoulder Assessment Form, patient self-report section. BMC Musculoskelet Disord.

[20] Richards R.R., An K., Bigliani L.U., Friedman R.J., Gartsman G.M., Gristina A.G. (1994). A standardized method for the assessment of shoulder function. J Shoulder Elbow Surg.

[21] Roddey T.S., Olson S.L., Cook K.F., Gartsman G.M., Hanten W. (2000). Comparison of the University of California–Los Angeles Shoulder Scale and the Simple Shoulder Test with the Shoulder Pain and Disability Index: Single-administration reliability and validity. Phys Ther.

[22] Roy J.S., MacDermid J.C., Woodhouse L.J. (2009). Measuring shoulder function: a systematic review of four questionnaires. Arthritis Rheum.

[23] Themistocleous G.S., Goudelis G., Kyrou I., Chloros G.D., Krokos A., Galanos A. (2006). Translation into Greek, Cross-Cultural Adaptation and Validation of the Disabilities of the Arm, Shoulder, and Hand Questionnaire (DASH). J Hand Ther.

[24] Vrotsou K., Cuellar R., Silio F., Rodriguez M.A., Garay D., Busto G. (2016). Patient self-report section of the ASES questionnaire: a Spanish validation study using classical test theory and the Rasch model. Health Qual Life Outcomes.

[25] Ware J.E., Sherbourne C. (1992). The MOS 36-items short-form survey (SF-36): I. Conceptual framework and items selection. Med Care.

[26] Yahia A., Guermazi M., Khmekhem M., Ghroubi S., Ayedi K., Elleuch M.H. (2011). Translation into Arabic and validation of the ASES index in assessment of shoulder disabilities. Ann Phys Rehabil Med.

